# Characterizing and demonstrating the role of *Klebsiella* SSN1 exopolysaccharide in osmotic stress tolerance using neutron radiography

**DOI:** 10.1038/s41598-023-37133-w

**Published:** 2023-06-21

**Authors:** Sheetal Sharma, Tushar Roy, Yogesh Kashyap, Martin Buck, Jorg Schumacher, Dweipayan Goswami, Shraddha Gang, Meenu Saraf

**Affiliations:** 1 Dept. of Microbiology and Biotechnology, School of Sciences, Gujarat University, Ahmedabad, 380009 India; 2 Technical Physics Division, Bhabha Atomic Research Centre, Trombay, Mumbai 400085 India; 3 Department of Life Science, Faculty of Natural Sciences, Imperial College, London, SW7 2AZ UK

**Keywords:** Applied microbiology, Environmental microbiology

## Abstract

Exopolysaccharides (EPS) are organic macromolecules naturally secreted by many microorganisms. EPS is increasingly used for agriculture and industrial purposes. This study focuses on isolate *Klebsiella* *pneumonia* SSN1, *Klebsiella quasipeumonniae* SGM81 isolated from rhizosphere to explore its water retention efficiency under drought conditions. Neutron Radiography was used to visualise water distribution in the sand under normal and drought conditions in the presence and absence of EPS producing bacteria. The EPS production was studied by applying Box Behnken design (BBD) under drought stress which was artificially induced by using polyethene glycol 6000 under osmotic stress condition 3.65% w/v of EPS dry weight was obtained. The relative water content (RWC) is used to calculate the amount of water present in the sand and was further studied by Neutron Radiography imaging with appropriate controls. FTIR and HPLC were also carried out for the characterisation of the extracted EPS. The sand experiments revealed that after 24 h of evaporation, the highest RWC was maintained by SSN1 at 29.7% compared to SGM81 (19.06%). SSN1 was found to release L-arabinose as the main sugar of its EPS under drought stress conditions by HPLC method. The FTIR data indicated the presence of β-glucans and polysaccharide α-pyranose between wavenumber 700 cm^−1^–1500 cm^−1^ and 1017 cm^−1^–1200 cm^−1^ respectively. The HPLC characterization of extracted EPS from osmotic stressed cells (run 3) displayed a peak designated to L-arabinose at 10.3 retention time (RT) for 132.4 mM concentration. While from run 5 with the controlled condition indicated the presence of L-rhamnose at 7.3 RT for 87 mM concentration. Neutron radiography enables the visualisation of water distribution in the sand as well as water transport in root-soil systems in situ. SSN1 has elicited EPS production in drought conditions with a low level of nitrogen and carbon.

## Introduction

Environmental changes are strongly influenced by anthropogenic activities which result in abiotic stresses in the soil^1,2^. Climate variability leads to unfavourable conditions like drought. Extreme temperatures and heat waves are experienced in many parts of the world. This can have a direct or indirect impact on the agriculture sector as well as the economy of the particular country. The consequences of drought on the agriculture sector can lead to an economic loss in the country^3,4^. Deficiency of precipitation, hike in evaporation and transpiration rate and proliferation of water resources, are the fundamental parameters that lead to drought. Another reason for the increase in drought frequency is the discharge of groundwater and scarcity of rainfall which ultimately lead to drought conditions in many parts of the world^5^. After the subsequent water losses and deep percolation, the water thereafter which remains in the soil for uptake by plants and soil microbiota is termed green water^6^. Worldwide around 90% of green water is consumed for agricultural purposes^7^. The cumulative amount of productive green water flow depends on the correlation between the growth of plants in the root section and soil microbiota and transpiration rate^8^. Renewal of soil nutrients and enhanced production of plant growth by plant growth promoting rhizobacteria has been an interesting area for decades examined at a distinct level in various parts of the world^9^. Various biotic movements in the soil ecosystem involve PGPR mechanisms to increase yield and feasible growth of crop management^10^. One such mechanism is the biosynthesis of exopolysaccharides. It plays a major role in conserving the water content of plants and maintaining soil moisture^11^. Bacteria like *Azotobacter vinelandii, Bacillus drentensis*, *Enterobacter cloacae*, *Agrobacterium sp*., *Xanthomonas sp*. are EPS producing PGPRs vital in the renewal of soil fertility^12^. EPS play a significant role in maintaining relative water potential, and aggregation of soil particles. It also establishes contact between root architecture and rhizobacteria directly under abiotic stresses like dry weather and water scarcity conditions^13^.

EPS released by bacteria may have utility in increasing agricultural output, although this has not been fully investigated. Alginates, which are widely known for their industrial applications, are widespread in EPS^14,15^. It can be used as food, material for wound treatment, to immobilise enzymes through entrapment, and to make artificial seeds in plant tissue culture. EPS released by bacteria is important in the encystment of synthetic seeds, as it protects from water deficit and predation by protozoan phages^16^ and it also affects the entry of anti-microbial compounds^17^ and toxicants metals^18^. The wider applications of polymeric substances have been explained by Honarkar et al. and Aravamudhan et al.^19,20^. Natural biopolymers are used in a variety of applications, including bone tissue engineering, drug targeting, drug carrier, prosthetics, hydrogels, filtration membranes, food packaging, and pharmaceutical preparations, depending on their particular features. Synthetic polymers lack the desired features of safety, bioactivity, biocompatibility, and biodegradability when compared to biopolymers. As a result, biopolymers play an essential role in a variety of fields.

The most recent application to study water retention in the plant and soil is by using Neutron radiography. The neutrons display maximum interaction with protons rather than other nuclei, which consecutively makes hydrogen^21^ containing material appear highly visible^22^. Neutron imaging is ideal for agricultural research applications because neutrons are sensitive to hydrogen, which the plants or roots contain in abundance. In contrast, the sand does not have water-holding characteristics which reduce the amount of hydrogen. That means the plant roots show up as dark, clear, and distinct regions in the radiographic images. Neutrons are ideally suited for this task since they readily penetrate most common materials but are strongly attenuated by those containing hydrogen such as water. Also, water distribution in soil and roots can be easily imaged and quantified. One of the leading concerns in agriculture is the decreasing availability of water for irrigation leading to drought-like conditions. Various methods are being devised to increase soil water retention. One such method is the use of EPS producing bacteria which is the subject of this work. The high neutron attenuation coefficient of hydrogen (in water) makes neutron radiography highly potential for studying or measuring soil water dynamics. The objective of the present experiment is to use neutron radiography to directly visualize water retention/distribution in the soil during evaporation in the presence and absence of EPS producing bacteria^23–25^.

The present study deals with the extraction and qualitative analysis of EPS through the modernistic NR technique. Our research investigated the water retention capabilities of exopolysaccharides (EPS) derived from three distinct Klebsiella strains, namely *Klebsiella pneumoniae* SSN1, *Klebsiella quasipneumoniae* SGM81, and *Klebsiella oxytoca* M5a1. Another significant aspect of our study was the quantification of EPS in artificial stress condition. PEG is used to induce drought condition as it lowers the osmotic potential of the solution. Shahzadi Mahapara^26^ indicate that the use of PEG for artificially inducing osmotic stress in the medium is a reliable approach to mimic the abiotic stress Here, the optimization of EPS production by SSN1 was studied in the presence and absence of polyethylene glycol 6000 with the application of a Box-Behnken design tool.

## Material and methodology

### Bacterial strain and growth media

Experiments were conducted with three different *Klebsiella* strain *Klebsiella pneumonia* SSN1*, Klebsiella quasipneumoniae* SGM81, and *Klebsiella oxytoca* M5a1. SSN1 was isolated from the rhizosphere of barley near the Dholka region of Gujarat, India, SGM81 was isolated from the rhizosphere of *Dianthus caryophyllus* and M5a1 was obtained from Martin Buck and Jorg Schumacher, Imperial College London. The whole genome sequencing data for SSN1 (PRJNA643013) indicates the absence of a pathogenic gene, and thus SSN1 can be used for practical purposes. SGM81 was selected as a positive control for EPS production, whereas *Klebsiella* M5a1 lacked EPS production ability, therefore used as a negative control for all the experiments. The EPS production in SSN1 and SGM81 was carried out using Jensen nitrogen-free media (JNFM) with modification in sucrose concentration and the addition of peptone as a nitrogen source. The media consist of sucrose 3% w/v, K_2_HPO_4_ 0.1% w/v, MgSO_4_ 0.05% w/v, NaCl 0.05% w/v, FeSO_4_ 0.01% w/v, Na_2_MoO_4_·2H_2_O 0.0005% w/v, CaCO_3_ 0.2% w/v and peptone 1% w/v.

### Activation and growth curve of bacteria

Initially, screening was conducted in Jensen media with the addition of 1% w/v peptone as a nitrogen source in a 100 mL flask for each strain SGM81, M5a1 and SSN1. Bacterial cells were harvested by centrifugation at 5000 rpm for 10 min from overnight grown JNFM media and suspended in N-saline to achieve the optical density (OD) of 0.1. Further, all the experiments were conducted in triplicates for every strain. This 1 mL bacterial suspension was inoculated in 100 mL JNFM and incubated at 26 °C ± 2 °C in a rotary shaker at 140 rpm. 10 mL of culture broth from each flask were collected at intervals of 24, 48, 72, 96, 120, 144 h and centrifuged at 10,000 rpm for 10 min to collect the cell biomass. The collected cell biomass was vortexed and washed twice with sterile N- saline and centrifuged again to collect pure biomass. The collected biomass was resuspended in 10 mL N- saline for spectrophotometric observation at 600 nm. All the observations were recorded in triplicates and their descriptive analysis was done by the statistical tool GraphPad Prism 8.

### Purification and quantification of EPS

Purification of EPS was carried out after 4 days following the solvent extraction method as discussed by Castellane et al. (2017)^27^. The broth was centrifuged at 10,000 rpm for 15 min at 4 °C to separate biomass pellet and supernatant. To this collected supernatant, a double volume of previously chilled 80% of acetone was added and kept overnight at − 20 °C to obtain EPS precipitates. The precipitates were filtered out with Whatman filter paper no 1 on day 2. The EPS extracted was subjected to a drying procedure in a hot air oven at 60 °C until completely dried. . Aggregated dry EPS was quantified using the formula: EPS (g% w/v) = W_2_ − W_1_/ V as mentioned in Shukla et al. (2021)^28^. Where W_2_ is the weight of an aluminium dish with the sample, W_1_ is the initial weight of an empty aluminium dish without a sample, and V is the volume of the sample.

### Optimization of EPS production by Box- Behnken design for Klebsiella SSN1

The Box Behnken design with fifteen different media compositions was employed to study the optimization of EPS production^29^. All the experimental set-ups were conducted for SSN1 only (based on the highest EPS producing ability) and in replicates of three. The highest, median and lowest (+ 1, 0 − 1) range was selected for three different factors as displayed in Table 1. Sucrose, peptone and calcium carbonate were selected as carbon (F1), nitrogen (F2) and macro element (F3) respectively in the media as mentioned in Table 2. To investigate the effect of stress on EPS production, 20% PEG 6000 was added to each flask. All the designated flasks were designed in two sets as control and osmotic stress. The resulting dry weight of EPS obtained by both treatments was denoted as R1 for control and R2 for osmotic stress conditions. The designated flasks were inoculated with 1 mL of the young culture of SSN1, having a CFU count of 10^9^ and incubated at 26 °C ± 2 °C for 144 h. The statistical analyses were carried out by the State ease Design expert (version 12), including one-way ANOVA and response surface methodology. EPS was extracted from each flask and calculated for dry weight production as described in the previous Section “Purification and quantification of EPS”. A designated flask showing excess EPS dry weight was further selected for CFU count to correlate bacterial cell number and enhance EPS production with peptone.

**Table 1:** Factorial design of box Behnken represented.

Factor	Variables in g% w/v	Minimum	Maximum	Coded low	Coded high
A	Sucrose	1.0000	5.00	− 1 ↔ 1.00	+ 1 ↔ 5.00
B	Peptone	0.1000	0.5000	− 1 ↔ 0.10	+ 1 ↔ 0.50
C	CaCO3	0.1000	0.5000	− 1 ↔ 0.10	+ 1 ↔ 0.50

**Table 2:** BBD representing three independent variables and their responses in normal and stress condition.

Std	Run	Factor 1	Factor 2	Factor 3	Response 1 (normal condition)	Response 2 (stress condition)
		A:Sucrose	B:Peptone	C: CaCO_3_	Control	PEG 20%
		g% w/v				
12	1	3	0.5	0.5	1.42	1.62
8	2	5	0.3	0.5	2.68	2.95
4	3	5	0.5	0.3	1.48	3.65
5	4	1	0.3	0.1	1.25	2.98
2	5	5	0.1	0.3	3.57	1.26
1	6	1	0.1	0.3	0.98	3.25
3	7	1	0.5	0.3	2.62	2.29
9	8	3	0.1	0.1	1.13	0.2
10	9	3	0.5	0.1	1.52	1.25
13	10	3	0.3	0.3	1.68	2.36
6	11	5	0.3	0.1	0.25	2.74
7	12	1	0.3	0.5	0.59	2.96
11	13	3	0.1	0.5	2.36	1.08
14	14	3	0.3	0.3	2.5	2.68
15	15	3	0.3	0.3	2.4	2.54

### Neutron radiography experiment

The Neutron radiography experiment was performed using thermal neutrons at the Advanced Neutron Imaging Beamline at Dhruva research reactor, Bhabha Atomic Research Centre, India. The thermal neutron flux at the sample was 4 × 10^7^ n/(s-cm^2^) with the L/D ratio (collimation ratio where L is the length from the input aperture of the collimator to the sample and D is the diameter of the input aperture) of 160. The images were recorded using a detector set-up consisting of a ^6^LiF/ZnS scintillator, mirror, lens and sCMOS camera. The field-of-view for the present experiment was ~ 70 mm X 70 mm with a spatial resolution of 100 µm per pixel.

For the experiments, soil columns were prepared in four identical cuvettes (10 mm × 10 mm × 50 mm) with finely sieved sterile sandy soil samples. 1 mL of water was added uniformly to the control sample. For the remaining three samples, 0.5 ml of water and 0.5 ml of EPS broth of SGM81, M5a1, and SSN1, were added respectively. All four saturated soil columns were placed in a row and exposed to the neutron beam for radiography. All samples were imaged simultaneously to ensure the same environmental conditions. Each neutron radiography was acquired for the 20 s. A series of individual radiographs were acquired over a total duration of approximately 25 h. The images were normalized using flat-field correction to minimize homogeneities due to fluctuations in beam intensity.

In another series of experiments, the relative water content of sand was studied by growing a chickpea plant sample and treating them with EPS extracted with SSN1. In this experiment, aluminium pots of 2 cm in diameter and 10 cm in length were used to cultivate chickpeas. For 1 min, seeds were surface sterilised with 70% sodium hypochlorite and rinsed with sterile distilled water. EPS production media were used to inoculate the SSN1 strain. The culture broth was collected after reaching an exponential phase so that the optical density at 600 nm could be adjusted to a constant of 0.1. After two minutes of bacterization, the seeds were placed in an aluminium pot filled with sterile sand. The pots were irrigated with water for 21 days before being sent to BARC for analysis of NR. The samples were again irrigated with 1 mL of water for control and 1 mL of EPS broth in their respective pots. NR analysis was done for 3 days at an interval of every 24 h^25^.

The flat field correction is done by acquiring neutron radiographs of the same exposure time without the sample in the beam (open-beam image *I*_*open*_) and with the beam shutter closed (dark-field image *I*_*dark*_). The flat field correction is then defined as: I _flatfield_ = I–I_dark_/I_open_–I_dark_, where I is the raw image intensity. A 3 × 3 spatial median filter is then applied to the flat field image to remove shot noise.

### Structural characterization of EPS production by Klebsiella SSN1 using FTIR

The functional group differential pattern was identified by FTIR. The molecular curve produced by SSN1 in control and stress conditions was compared by the FTIR spectrum, ranging from 400 cm^−1^ to 4000 cm^−1^ at 64 scans^30^. The extracted EPS was vacuum dried with liquid nitrogen and homogenized into fine powder to protect proteoglycans, lipids and structural components present in a sample. These samples were directly exposed to the FTIR (Bruker Alpha FTIR spectrometer) for the interpretation of the difference in the spectral range of control and stress conditions. The homogenized powder of dried EPS was dissolved in 1 mL of Milli-Q water and quantified for total carbohydrate and total protein content. The phenol sulphuric method with slight modification was used for estimating the total carbohydrate content of EPS^31^. 100 µL of 80% phenol solution was added to 200 µL of a sample, followed by 1 mL of concentrated H_2_SO_4_. This mixture was settled for 10 min at room temperature to avoid acid bubbles. The final concentration of EPS was measured spectrophotometrically at 490 nm. Total protein was quantified by the Bradford method. Briefly, 30µL ofthe sample was mixed with 1 mL of Bradford reagent and rested at room temperature for 5 min. Proteins were quantified by comparing with standard BSA (0.0–0.80 µg/mL) at 595 nm^32^.

### Molecular characterization of EPS production by Klebsiella SSN1 using HPLC

To determine the monomeric sugar group, we conducted an HPLC analysis, as reported in Result Table 2. Here, the EPS extracted under control and osmotic stress conditions from runs 3 and 5 were quantified for molecular characterization. We dissolved 2 g of the extracted EPS from each sample in double the volume of 2 M H_2_SO_4_, followed by hydrolysis in a water bath at 100 °C for two hours. The solution was then neutralized with 1 M Na_2_CO_3_. For comparative purposes, we prepared standard sugar solutions at a concentration of 100 mM for all the monomers. These included Sigma-grade L-rhamnose, glucose, galactose, trehalose, L-arabinose, mannose, and sucrose. We performed the HPLC analysis on a Shimadzu HPLC system, using a synchronic amino column and a Refractive Index (RI) detector 20-A. The flow rate was set at 1.0 mL/min, with an acetonitrile: MilliQ-water (70:30) mobile phase and a column temperature of 30 °C. We injected 20 µL of both the sample and the standard for the analysis^33^.

## Results

### Growth pattern and EPS production

**Figure 1:**
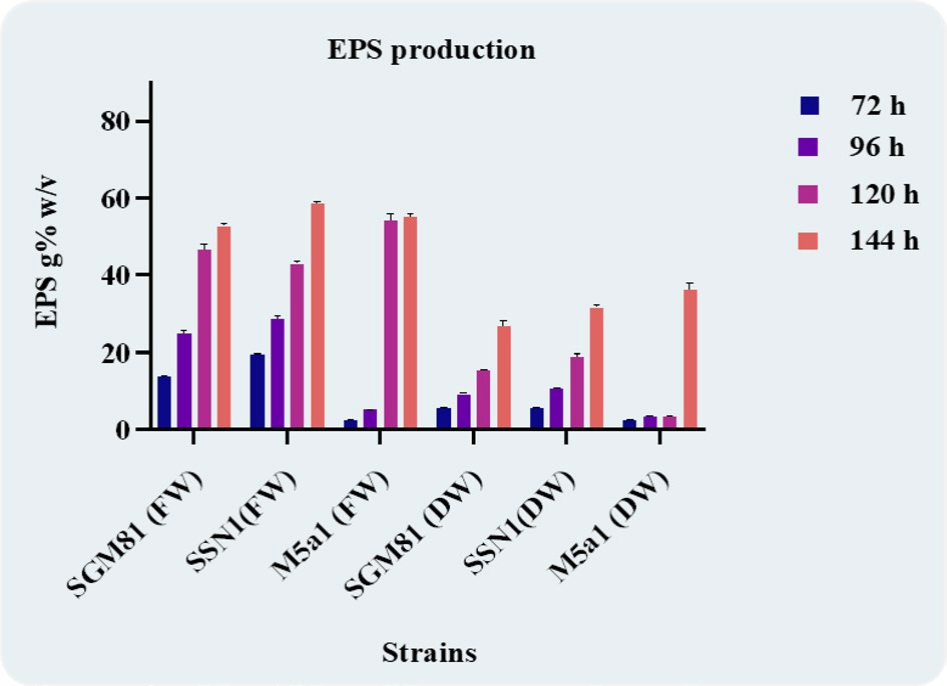
EPS production by selected isolate in correlation to incubation hours. The FW indicates the fresh weight after extraction and DW indicates the Dry weight of EPS after the drying process. SGM81 and SSN1 were representing identical results, the EPS production increased with incubation time, while M5a1 was representing a linear graph depicting there was no EPS production.

Peptone as a nitrogen source and sucrose as a carbon source has been investigated for their effect on EPS production. To evaluate the role of cell density in EPS production, the optical cell density of bacterial cells was studied with reference to incubation hours. It was observed that the increase in cell density broadens the range of EPS in production media as shown in Supplementary Fig. 1. The maximum optical density (OD) for SSN1 was observed to be 0.35 after 24 h and increased to 1.95 after 144 h. During a 72-h incubation period, the OD of SGM81 reached 0.70, slightly higher than the 0.62 recorded for SSN1. From the fourth day onward, the cultures producing EPS demonstrated increased broth turbidity, following the order M5a1 < SGM81 < SSN1. . Maximum growth was recorded after 144 h for SSN1 (2.10) and SGM81 (1.95) whereas, there was no significant increase for M5a1 (1.2). To reveal the patterns of EPS production in response to incubation time, EPS was extracted from each flask by solvent extraction method with prechilled 80% acetone. This extracted EPS was initially quantified for total fresh weight and later for total dry weight at an interval of 24 h. The fresh weight of extracted precipitate from SGM81 was 13.6 g% w/v on day 3 to 22.5 g% w/v on day 5. There was a significant increase in the dry weight of extracted precipitate from SGM81 ranging from 13.6 g% w/v to 28.0 g% w/v following six days of the incubation period. The increase in fresh weight of EPS precipitates by SSN1, from 72 to 144 h was 19.8 g% w/v to 58.1 g% w/v as shown in Fig. 1. Between 3 and 6 days EPS from SSN1 increased from 8.6 g% w/v to 31.0 g% w/v. However, there was no increase in EPS concentration in M5a1.

### Optimization of EPS production using BBD

The BBD based on the RSM technique was used to standardize media composition with carbon, nitrogen and calcium sources for EPS production. The design model was run for control and stress conditions in which 20% PEG 6000 was added as a supplement to increase drought stress artificially. There were 15 runs for each study consisting of the lowest to the highest value of all three factors.

The model consists of 15 flasks with distinct media compositions Table 2**.** Further statistical analysis was done using ANOVA and Fit statistic for the quadratic polynomial model after obtaining the dry weight of EPS of Response1 (R1) and Response 2 (R2). Our designed model was found significant for both responses and the model showing P values less than 0.0500 was considered significant model terms illustrated in Table 3. R1 was showing significant model terms for A, C, AB, AC, C^2^ and R2 was found significant for B, AB, A^2^, B^2^, and C^2^. The predicted R^2^ and adjusted R^2^ for the response R1 was 0.8239, 0.8843 whereas for R2 obtained predicted R^2^ was 0.7914 and the adjusted R^2^ was 0.9539. For R1 without drought, the “Lack of Fit F-value” was 0.9440 and the value implies 0.3393 for R2 under drought conditions which indicates Lack of fit was insignificant for both responses and related to the pure error.

$$ {\text{Control}}:\, - \,{1}.{56698}\, + \,0.{\text{613125 X1}}\, + \,0{\text{598125X2}}\, + \,{1}0.{\text{18125 X3}}\, - \,{2}.{\text{33125 X1X2}}\, + \,{1}.{\text{93125 X1X3}}\, - \,{8}.{3125}0{\text{ X2X3}}\, - \,0.0{\text{55729 X1}}^{{2}} \, + \,{4}0{8}0{2}0{\text{8X2}}^{{2}} \, - \,{\text{1944792X3}}^{{2}} , $$

$$ {\text{PEG 2}}0\% :\, + \,{2}.{\text{43198 X1}}\, - \,{2}.{\text{19313 X2}}\, + \,{9}.{\text{94375 X3}}\, + \,{1}0.{\text{38125 X1X2}}\, + \,{2}.0{\text{9375 X1X3}}\, + \,0.{14375}0{\text{ X2X3}}\, + \,0.{\text{244479 X1}}^{{2}} \, - \,{22}.0{32}0{\text{8 X2}}^{{2}} \, - \,{14}.{927}0{\text{8 X3}}^{{2}} , $$

Where X1 = Sucrose, X2 = Peptone, X3 = CaCO_3_.

**Table 3:** ANOVA for quadratic model (A) Response 1: Control and (B) Response 2: PEG 20%.

Response 1: Control (A)							Response 2: PEG 20% (B)						
Source	Sum of Squares	df	Mean Square	F-value	p-value		Source	Sum of Squares	df	Mean Square	F-value	p-value	
Model	10.88	9	1.21	12.89	0.0058	Significant	Model	12.74	9	1.42	33.22	0.0006	Significant
A-Sucrose	0.8064	1	0.8064	8.60	0.0325		A-Sucrose	0.0968	1	0.0968	2.27	0.1922	
B-Peptone	0.1250	1	0.1250	1.33	0.3005		B-Peptone	1.14	1	1.14	26.74	0.0036	
C-CaCO_3_	1.05	1	1.05	11.21	0.0204		C-CaCO_3_	0.2592	1	0.2592	6.08	0.0568	
AB	3.48	1	3.48	37.09	0.0017		AB	2.81	1	2.81	65.81	0.0005	
AC	2.39	1	2.39	25.45	0.0039		AC	0.0132	1	0.0132	0.3102	0.6016	
BC	0.4422	1	0.4422	4.72	0.0820		BC	0.0650	1	0.0650	1.53	0.2717	
A^2^	0.1835	1	0.1835	1.96	0.2208		A^2^	3.53	1	3.53	82.82	0.0003	
B^2^	0.1362	1	0.1362	1.45	0.2820		B^2^	2.94	1	2.94	68.92	0.0004	
C^2^	2.23	1	2.23	23.83	0.0045		C^2^	1.32	1	1.32	30.88	0.0026	
Residual	0.4689	5	0.0938				Residual	0.2132	5	0.0426			
Lack of Fit	0.0687	3	0.0229	0.1143	0.9440	Not significant	Lack of Fit	0.1617	3	0.0539	2.09	0.3393	Not significant
Pure Error	0.4003	2	0.2001				Pure Error	0.0515	2	0.0257			
Cor Total	11.35	14					Cor Total	12.96	14				

### Analysis of 3 residual plots

**Figure 2:**
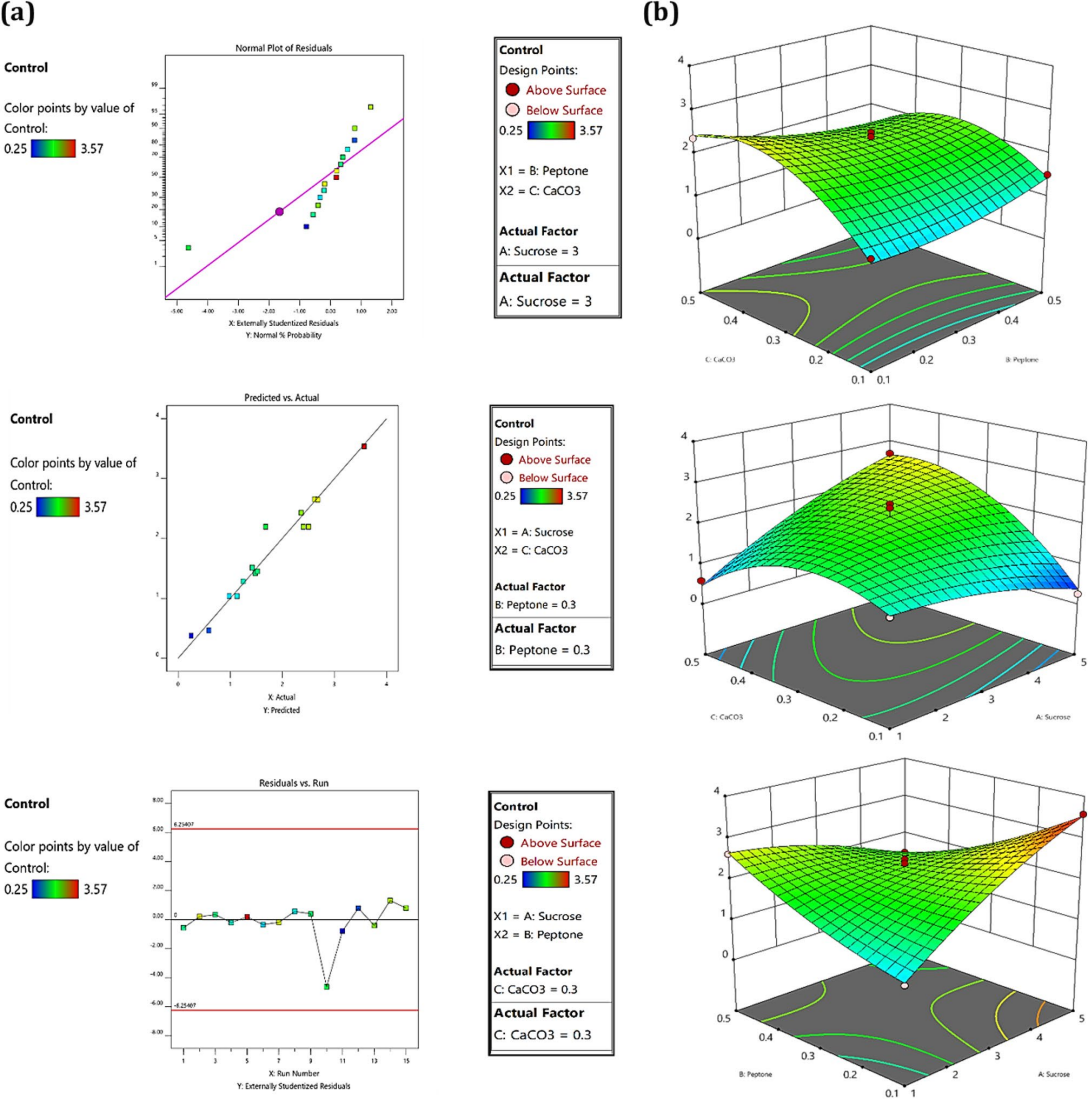
Results of BBD demonstrating 3D plot in control condition for all the three factors, (**a**) represents diagnosis analysis of 3D residual plot for verification of design and (**b**) represents the 3D graph of the response generated in a control condition.

**Figure 3:**
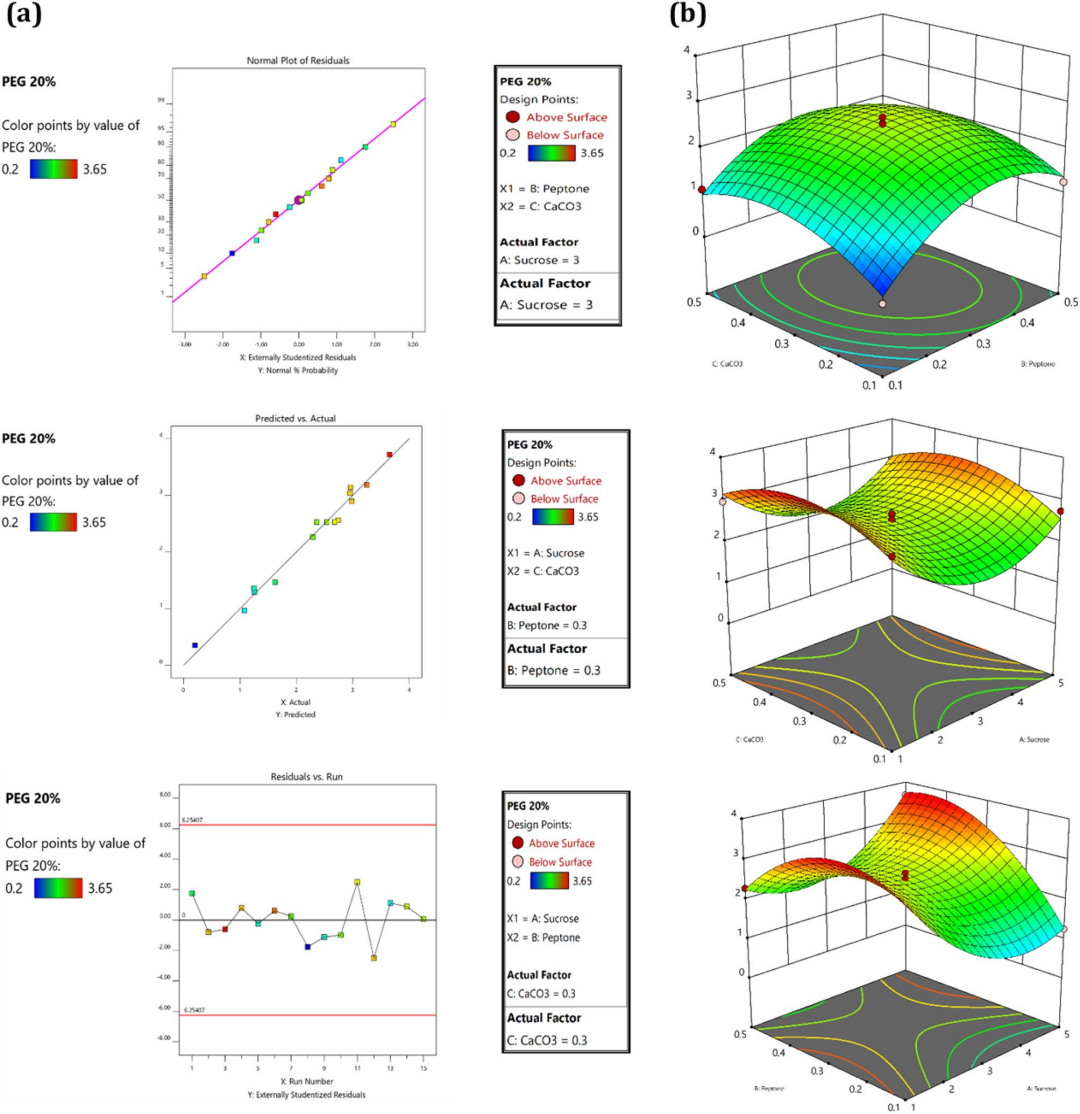
Results of BBD demonstrating 3D plot in osmotic stress condition for all the three factors, (**a**) represents diagnosis analysis of 3D residual plot for verification of design and (**b**) represents the 3D graph of the response generated in osmotic stress condition.

The verification of the model is very crucial when it comes to statistical analysis, this is achieved by different types of residual plots generated against the number of the run. Normal residual graphs were analysed to verify the normality of data in response to all three factors. The spots of anticipation and estimated value were falling on either the straight line of proximity or close to vicinity predicted the best normality of data. The R1 Fig. 2a was depicting a less normalization value compared to R2 which was interpreting the maximum distribution of data normally. Another residual plot comprises the actual and predicted value of the EPS obtained in response to both treatments. In this plot, the straight line demonstrates the predicted value whereas the square box represents the actual value obtained. A good agreement was demonstrated between predicted and actual values for both responses. The externally studentized residual versus experimental run plot demonstrates if any variable has interfered with experimental data analysis. The calculation of the externally studentized residual plays a crucial role in our analysis. Specifically, it aids in identifying any potential interference from variables within our experimental data analysis. By plotting the externally studentized residual against the experimental run, we are able to visualize and understand any discrepancies or outliers in our data that could impact our findings. The plot is the best fit for model consideration when it appears in scattered nature as shown in Fig. 3a.

### Factorial composition for EPS production by Response Surface Methodology graph

#### For R1

To investigate the independent effect of three variables in both responses, 3D graphs were created. We evaluated the minimum and maximum reciprocal action of three factors A (Sucrose), B (Peptone) and C (CaCo3) for EPS production. Initially, the 3D response was created for A and B with a Cconstant at 0.3% interpreting the effect of carbon and nitrogen on EPS production. In this case, the amount of EPS dry weight recovered was ranging from 0.98 g% w/v to 3.57 g% w/v. The maximum amount of EPS was recovered for 3.57 g% w/v when the sucrose (A) and peptone (B) concentration was 5% and 0.1% respectively. The results suggested an increase in sucrose and a decrease in peptone concentration can lead to maximum EPS production. The interrelation between sucrose and CaCO_3_ was evaluated when the value of B peptone was considered constant at 0.3% as shown in Fig. 2b. With an increase in the concentration of sucrose from 1 to 5% and CaCO_3_ from 0.1% to 0.5% EPS dry weight was increased from 0.25 g% w/v to 268 g% w/v. The maximum EPS was produced with 5% sucrose, CaCO_3_ 0.5% and peptone 0.3%. Most of EPS dry weight was recovered at 2.36 g% w/v of concentration when sucrose was constant at 3% and there was a decrease in the concentration of peptone 0.1% but an increase in CaCO_3_ level 0.5%.

### For R2

The activity of SSN1 in osmotic stress conditions was examined by adding PEG to create stress. It was significantly noted that stress conditions enhance the production of EPS. All the designated flasks include the same set of media compositions except for the addition of 20% PEG in each flask. To make sure that PEG is not acting as an additional carbon source in production media we have conducted two experimental sets which consisted of (a) 2% sucrose + 20 PEG % and (b) 0.5. peptone% + 20. PEG%. After incubation of 144 h, there was no EPS production was observed in both flasks, indicating that PEG is not acting as the additional carbon source in experiments conducted for osmotic stress conditions.

There were similar results obtained for sucrose and peptone when the CaCO_3_ concentration was constant at 0.3% (w/v). When sucrose level increases from 1 to 5% and peptone reached a maximum level of 0.5%, the amount of EPS dry weight increases from 1.26 g% w/v to 3.65 g% w/v as shown in Fig. 3b. The strength of peptone concentration in stress conditions on EPS production was observed by retaining peptone at 0.3%. The total dry weight of EPS was 2.98 g% w/v when sucrose and CaCO_3_ were at their minimal concentration of 1% and 0.1% respectively. This demonstrates that a decrease in the amount of sucrose and CaCO_3_ can enhance EPS dry weight. When all the three factors are at the optimal range (3, 0.5, 0.5), the dry weight recovered was 2.68 g% w/v.

### Water holding capacity of EPS producing cell cultures using neutron radiography

**Figure 4:**
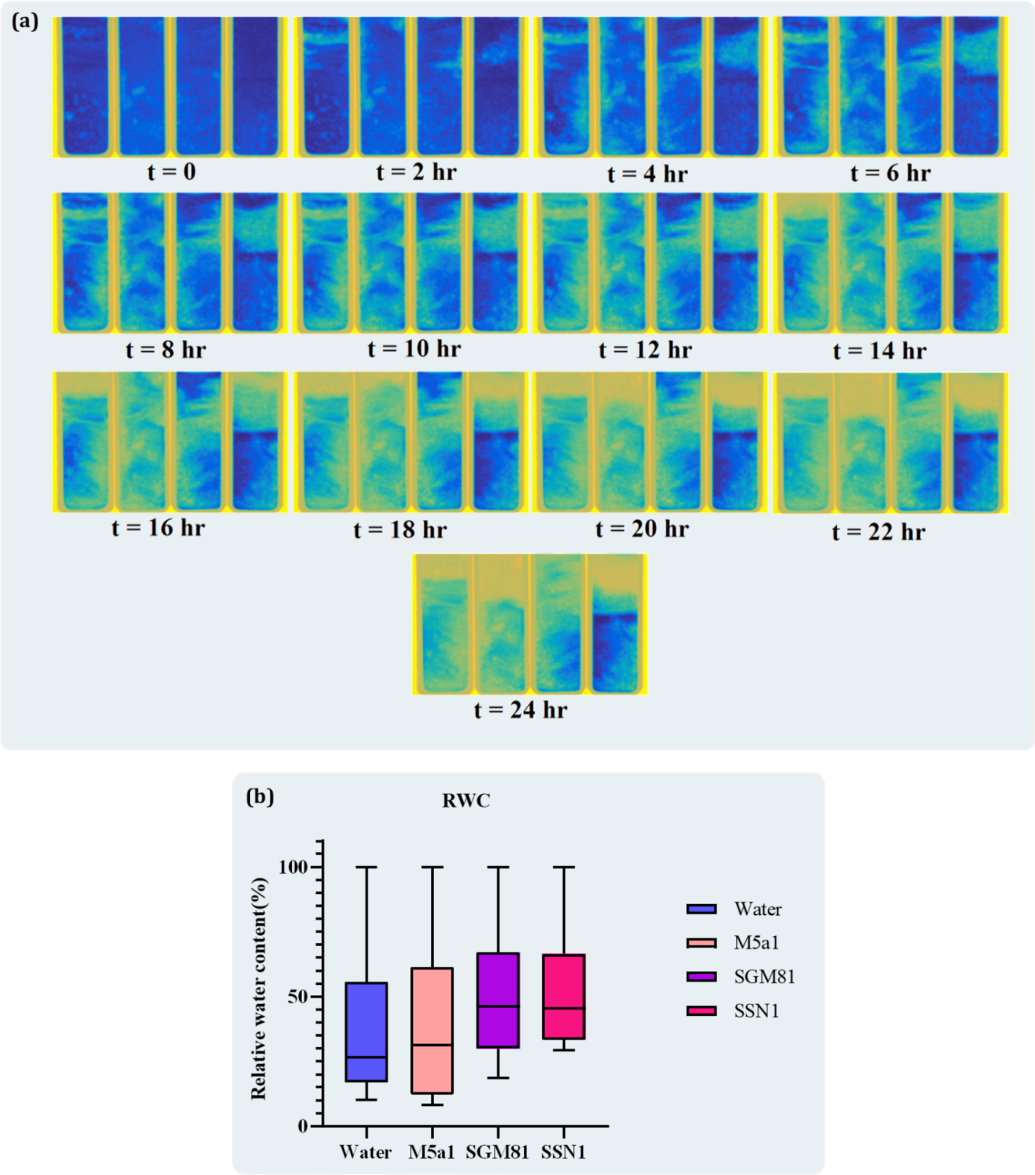
(**a**) Drying-out progression—Neutron radiographs at different time intervals. The samples are arranged as (from left to right) control (water), M5a1, SGM81, and SSN1 and (**b**) RWC of all the samples recorded during neutron radiography at an interval of every four hours. The RWC of M5a1 was almost similar to water, whereas SGM81 and SSN1 were showing maximum RWC ratio.

The RWC of each cuvette treated with SGM81, M5a1, SSN1, and water was calculated at an interval of every five minutes from 0 to 24 h continuously under ambient room temperature. The evaporation process of the sandy soil of different EPS treated and control samples was imaged. Supplementary Fig. 2 show the neutron radiographs (grayscale images) at t = 0 (start of experiment) and t = 24 h. The darker regions of the images correspond to more attenuation of the beam and hence higher water content. On the contrary, the lighter regions of the images correspond to less attenuation of the beam and hence lower water content. It can be observed that the evaporation/drying process is relatively slower in the samples treated with SGM81 and SSN1 whereas the sample treated with non-EPS strain M5a1 shows similar drying behaviour as the control sample. Figure 4 shows a sequence of radiographs at an interval of 2 h to elucidate the drying-out progression. The images have been pseudo-coloured for better visualization where water is represented by blue colour. The water distribution during the drying out process is not homogeneous, rather it progresses from top to bottom. This may be due to the reason that only the top surface of the sample is exposed to air during evaporation.

To quantify the variation in water content with time, we define the term Relative Water Content (RWC) as

$$ {\text{RWC}}\, = \, {\text{g}}\, - \,{\text{g}}_{{{\text{min}}}} \,/\,{\text{g}}_{{{\text{max}}}} \,{-}\,{\text{g}}_{{{\text{min}}}} \, \times \,{1}00\% , $$

where g is derived from following equation

$$ g = \int {\mu dx = \ln \left( {\frac{{I_{0} }}{I}} \right)} . $$

The terms *g*_*in*_ and *g*_*ax*_ correspond to a completely dry sand sample and a completely wet sand sample (sand saturated with water; condition at t = 0). For calculating *g*, the average intensity of pixels in the total area corresponding to each of the four samples is used from the neutron radiograph. In this way, we also account for the heterogeneous distribution of water in the sample during drying. The relative water content is normalized from 0 to 100%. Figure 4a shows a sequence of radiographs at intervals of 2 h to elucidate the drying-out progression. The images have been pseudo-coloured for better visualization where water is represented by blue colour. Figure 4b shows the variation of relative water content in different samples with time.

Initially, all the samples were showing 100% RWC of sand at 0 h. After 4 h of evaporation huge decline in RWC was recorded (water = 68.77%, M5a1 = 73.516%, SGM81 = 77.046%, SSN1 = 77.011%). EPS producing strains SSN1 (48.728%) and SGM81 (48.043%) appeared to have identical RWC until 12 of incubation. The evaporation rate was increased in samples treated with water and M5a1. After sixteen hours, RWC for SSN1 (36.411%) and SGM81 (36.35%) was similar whereas for water and M5a1 RWC was decreased by 80%. The highest RWC was maintained by SSN1 (29.7%) at the end of 24 h.

**Figure 5:**
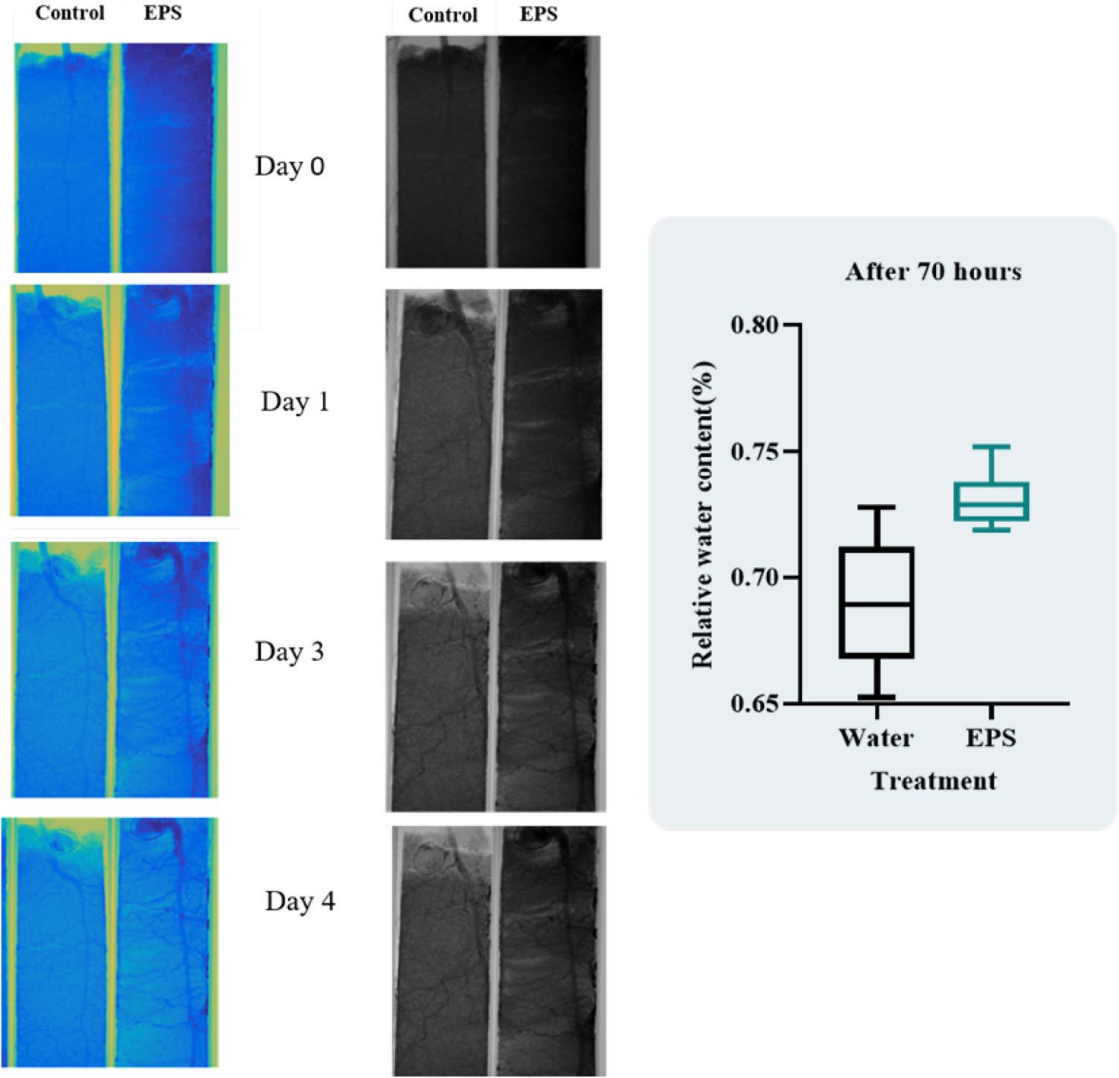
Plant sample treated with water shows less grey images representing minimum RWC of sand whereas chickpea plant treated with EPS was indicating more dark images. The RWC after 70 h is represented in graphical form, where EPS treated sample shows maximum RWC of sand after 3 days of the experiment.

The RWC content and root development of EPS treated plants was showing higher water retention capacity compared to the control pot. After 70 h of evaporation, the RWC of the EPS sample was 0.75% whereas for control was 0.65% as indicated in Fig. 5.

### Structural and molecular characteristics of EPS

The structural and functional groups present in monomers of extracted EPS were studied by the FTIR spectrum. Supplementary Fig. 3. The spectrum of purified EPS in the control condition was represented in the range of 647 cm^−1^ to 3950 cm^−1^, whereas the stress condition spectrum was recognized in the range of 704 to 3856 cm^−1^. A differential wave pattern was recorded for both samples, however, a contradictory wave band was represented between two samples. The infrared spectrum appearing between the range of 700—1500 indicates the presence of β-glucans in both samples (704 cm^−1^, 875 cm^−1^, 836 cm^−1^, 949 cm^−1^)^34^. The band between the range of 3051 cm^−1^–3950 cm^−1^ indicates strong stretching frequency for the O–H functional group in the non-stressed condition possibly indicating the presence of sugar amine or sugar acid. The FTIR spectra exhibited sharp absorbance of stretching at 3339.08 cm^−1^, while weak C–H stretching at 3.86.05 cm^−1^, 3810.57 cm^−1^, 3859.98 cm^−1^. We further observed sharp asymmetric C = O carboxylate at 1691.28 cm^−1^, C–C ring stretching at 1419.30 cm^−1^ and C–N stretching of a tertiary amine at 1243.77 cm^−1^. The region below 1500 cm called the fingerprint region interprets the position and bands of specific polysaccharides as mentioned by Chi et al.^35^. Peaks between a range of 1017 cm^−1^–1200 cm^−1^ were representing the presence of polysaccharide alpha pyranose. When the EPS was produced under osmotic stress conditions, the number of bands were comparatively low than the control sample. The structural moiety of polysaccharides was identical to the control wave pattern at wavenumber 3340.07 cm^−1^, while other spectra 1048.95 cm^−1^ and 1099.68 cm^−1^ were potentially indicating functional groups identical to β-glucans. The fingerprint region for stress conditions was less complex and with more monomers of sugars 949.83 cm^−1^, 875.33 cm^−1^, 836.99 cm^−1^, 704.64 cm^−1^. Spectrum representing protein amide was detected at 1239.76 cm^−1^ while this amide was lacking in control EPS.

The protein profile received for stress and control conditions also represented differences. Due to stress, there was an increased synthesis of protein in EPS as shown in Supplementary Table 1. For better understanding, two different runs were selected from the experimental design consisting of the lowest and highest value of peptone. In the control condition, the total protein content of extracted EPS was 0.9527 ± 0.03311% for run number 3 while in the stress condition there was a 78% increment. Similar results were obtained for runs 5 and 6 when peptone was at its lowest peak.

The EPS widely consists of different ranges of carbohydrates as shown in Supplementary Table 1. When the concentration of sucrose was highest in run 3 there was a significant decrease in carbohydrates by 93.58%. While in another example when the sucrose was at its lowest at 1%, the level of carbohydrates was increased in stress conditions compared to control. For the run 5 and 6 total sugar content was 16.67 ± 0.4410%, 19.50 ± 0.5774% but when the same set explored drought stress created by 20% PEG the level of carbohydrates in both samples were increased by 12.95% and 17.61.

**Figure 6:**
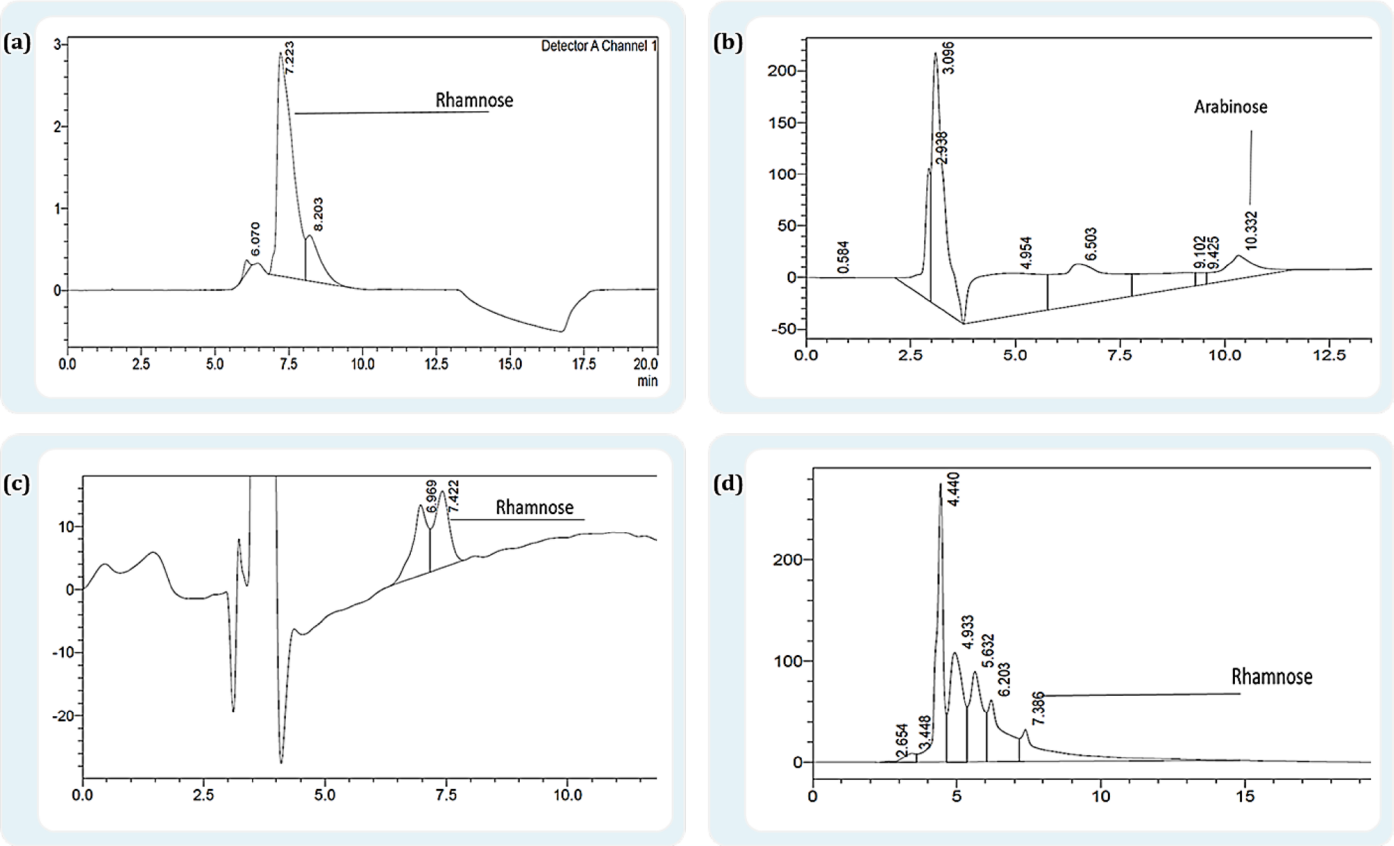
Chromatogram of extracted EPS for run 3 about BB design; (**a**) EPS extracted in the control condition was an indication peak for rhamnose, (**b**) EPS extracted in osmotic stress condition was showing a peak identical to arabinose. Chromatogram of extracted EPS for run 5 about BB design. Both (**c**) and (**d**) are indicating presence of rhamnose in control and stress condition.

The molecular characterization of EPS derivatives was carried out by HPLC. Samples were injected in a replicate of three to establish the retention time (RT). For run 3 in reference to BBD design EPS in a control, the condition was showing RT similar to L-rhamnose at 7.2 min Fig. 6a, whereas EPS treated with osmotic stress was indicating a peak similar to L-arabinose Fig. 6b. For run 5, a peak identical to L-rhamnose at 7.3 RT was observed in control and osmotic stress treated EPS as represented in Fig. 6c and d respectively. EPS extracted from run 3 was indicating the presence of L-rhamnose at 4.83 mM and L-arabinose at a concentration of 132.4 mM. Whereas EPS extracted in run 5 with the controlled condition was indicating the presence of L-rhamnose at.87 mM and 45.6 mM in the osmotic stress condition.

## Discussion

Sandhya and Ali^33^ suggested that the EPS composition of bacteria which is directly linked to the water holding capacity of *Pseudomonas putida* GAP-P45 under unfavourable conditions changes due to abiotic stress**.**

Bacterial EPS has many applications in different fields, one of the most interesting is in the agriculture sector to resist drought and water scarcity^36^. Bacterial EPS consists of many macromolecules, and lipids proteins that help to maintain the relative water content of the sand. Amao et al.^37^ isolated thirteen EPS producing bacteria from the cassava peel heap, among them four were identified as *Klebsiella* spp. The EPS secreted by *Klebsiella* spp. may have a potential role in agriculture productivity but only a few have been explored^38^.

To our knowledge, this is the first report on optimizing the production of EPS using Jensen media under PEG induced stress conditions*.* All three isolates were diazotrophic strains and our previously published article determines the best result with Jensen media and stress condition^39^. *Klebsiella* strain has been reported in the literature for EPS production but we have added significant value to work by inducing stress in EPS production media. PEG 6000 has been added as a supplement to induce drought stress artificially^40^. There were 15 runs for each study indicating consisting of the lowest to the highest value of all three factors. To evaluate the role of cell density in EPS production, growth patterns of bacterial cells were studied with reference to incubation hours. The Carbon/Nitrogen (C/N) ratio has always been in attention when it comes to EPS production media^41^. The earlier report supported many experiments indicating that the available carbon source fundamentally affects the concentration of EPS^42^. The *Klebsiella* HZ-7 strain used for EPS production with sucrose as a carbon source has reported 0.929 g % w/v at 1% of sucrose^21^, while SSN1 was producing 2.62 g % w/v without any stress. Experimental data reported by Sutharland (1982) and Ramírez-Castillo (2004)^43,44^ states that in case of carbon limitation there would be less release of by-products in EPS. Cui and Jia (2010)^45^ found that 12.6 g/L of peptone was best for EPS production from *Cordyceps militaris*; however, SSN1 was most productive at 0.1% peptone.

It was significantly noted that stress conditions enhance the production of EPS. So, to avoid any bias we have conducted one experimental set which consists of 2% sucrose + 20% PEG, 0.5% peptone + 20% PEG to make sure that PEG is not acting as an additional carbon source in production media. However, there was no EPS production observed in all those sets, indicating that PEG does not act as a carbon source to production media either solely or with sucrose. *Klebsiella* spp. has been of interest for decades for atmospheric nitrogen fixation activity^46^. SSN1 being a diazotrophic strain shows good EPS production under stress conditions even at 0.1% peptone.

Neutron radiography is another rapidly growing technique to study plant-soil interaction with the fascinating flow of neutrons in sample^24^. It is an excellent approach to studying interactions where hydrogen is sensitive to neutrons and creates contrast images in response to the presence of water. We have applied this technique to study the RWC and water holding capacity of EPS in the sand. Zheng et al.^5^ reported the application of EPS producing *Bacillus* spp in measuring soil water retention capacity with the help of neutron radiography in three different types of sand. We have studied the water holding characteristic of extracted EPS in soil. An hourly decrease in the water content of sand has been demonstrated in the form of video in Supplementary data 4. When EPS extracted from SSN1 was subjected to sand at ambient temperature for 24 h, it was noticed that the water retention capacity of cuvette treated with EPS was increased by 55.82% in comparison to SGM81 which was considered a positive control for experiments. Maximum water loss was observed when sand was treated with M5a1 and water. When EPS broth was treated with chickpea plant the water retention capacity was enhanced by 70% compared to untreated pot. The earlier experiment conducted by Zheng et al. (2018) indicated the same results for sand treated with EPS producing bacterial culture^5^.

EPS causes the aggregation of sand particles and increases the water holding capacity due to the presence of dense and mucoid material, however, there was no such interaction observed when M5a1 broth was added to the sand. The FTIR spectrum was used to compare the bending and vibration of sugar moieties created due to stress^47^. Structural characterization detected in the dried EPS sample was compared with reported data of *Klebsiella Oxytoca*^37^. There were multiple peaks observed in the control sample which indicates the presence of some complex monosaccharide, 1796.19 cm^−1^, 1755.74 cm^−1^ was assigned for the presence of glycolic acid and diethyl-ester. Only a few assigned amides in the designated wave was observed in the EPS sample treated with PEG, which indicates the presence of proteins in EPS and enhances the binding affinity of polysaccharide with metallic cations^48^. The HPLC analysis of all the samples indicated the presence of rhamnose in the control condition whereas only arabinose was secreted in osmotic stress. Serrat et al. (1995)^49^ reported *Klebsiella* sp I-714 for the production of L-rhamnose from bacterial EPS with the minor difference for the LPS associated sugar characterization in all the samples.

## Conclusion and future implications

In the present work, the structural and molecular characteristics of *Klebsiella* EPS were studied providing varied concentration of carbon–nitrogen sources. EPS consist of many beneficial macromolecules which needs further investigation. We have explored the use of neutron radiography, a non invasive tool for researching water transport in root-soil systems in situ. This is the first study where optimization of media was carried out in drought conditions. In summary, SSN1 has elicited EPS production in drought conditions with a low level of nitrogen and carbon. Also, SSN1 was able to maintain the water retention in the sand at 29.7% compared to non-EPS producers. A trial conducted with chickpea plants has presented significant results when treated with EPS, however, more experiments could be done in future to clear the role of EPS in stress conditions. Although *Klebsiella* can be an opportunistic pathogen as well as a soil dwelling non pathogen, its extracted EPS can act as a beneficial additive along with PGPR for drought tolerance, and accumulation of nutrients. Further field studies must be carried out to explore the full potential of SSN1 to be used as a soil conditioning agent. Plant tomography could also be performed to understand water flow in the root-soil-microbe system while focusing on different plant types along with model plant *Arabidopsis thaliana.*

## Supplementary Information

Supplementary Video 1.

Supplementary Information 1.

## Data Availability

The datasets used and/or analysed during the current study are available from the corresponding author upon reasonable request.

## Abbreviations

PGPR: Plant growth promoting rhizobacteria
EPS: Exopolysaccharide
BBD: Box-Behnken design
PEG: Polyethylene glycol
JNFM: Jensen nitrogen free media
Response 1: Flask treated with no stress (control)
Response 2: Flask treated with osmotic stress condition (test)
