# Supplementary figures and images for: Characterizing and demonstrating the role of Klebsiella SSN1 exopolysaccharide in osmotic stress tolerance using neutron radiography
Source: Sci Rep. 2023 Jun 21;13:10052. doi: 10.1038/s41598-023-37133-w (PMC10284798; doi:10.1038/s41598-023-37133-w)

## Slide 1
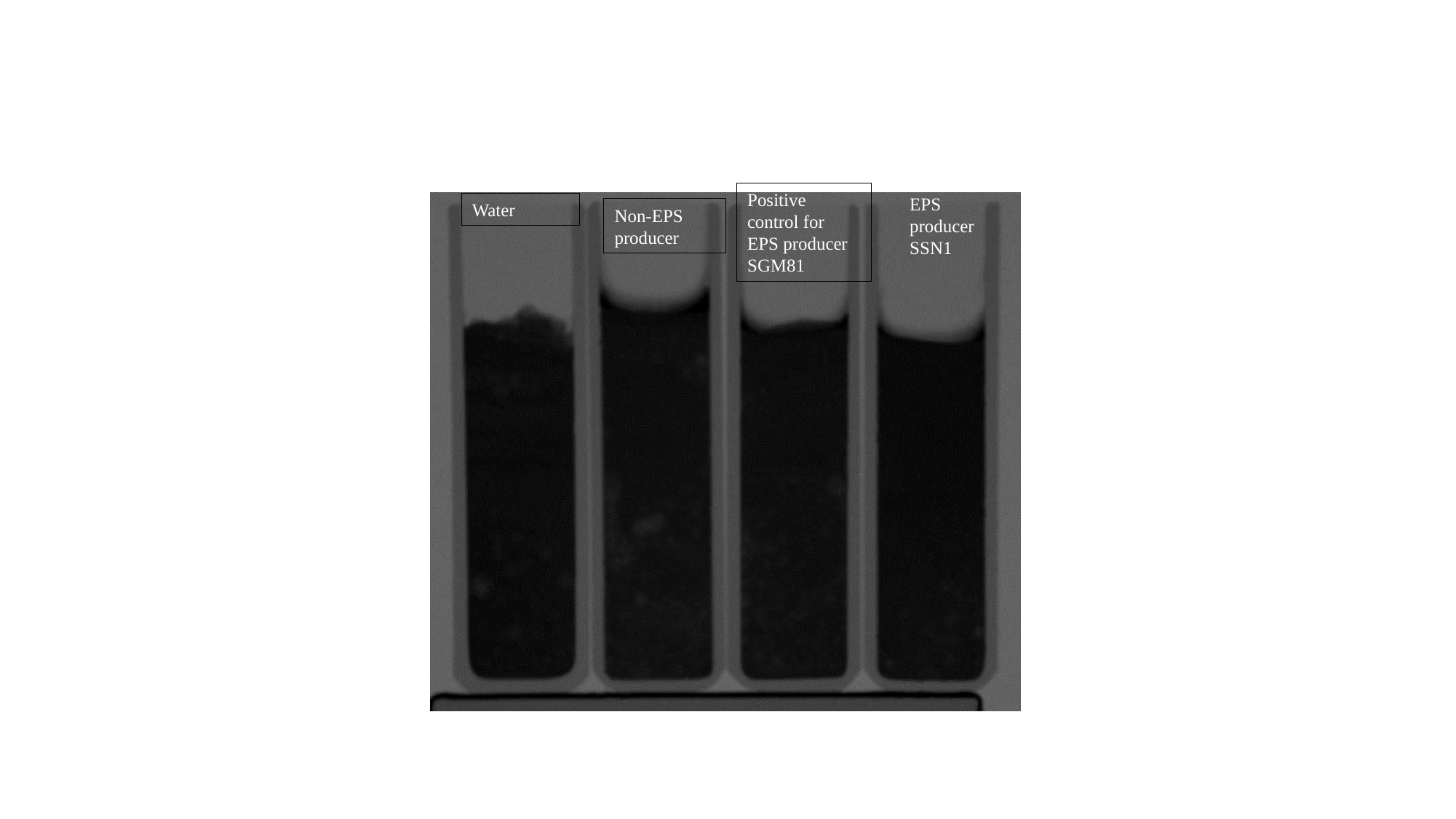

Positive control for EPS producer SGM81
EPS producer SSN1
Water
Non-EPS producer

Supplement: Supplementary file 1 — Supplementary Video 1. [file 41598_2023_37133_MOESM1_ESM.pptx]
