# Supplementary material for: Characterizing and demonstrating the role of Klebsiella SSN1 exopolysaccharide in osmotic stress tolerance using neutron radiography
Source: Sci Rep. 2023 Jun 21;13:10052. doi: 10.1038/s41598-023-37133-w (PMC10284798; doi:10.1038/s41598-023-37133-w)
Supplement: Supplementary file 2 — Supplementary Information 1. [file 41598_2023_37133_MOESM2_ESM.pdf]

Supplementary Material

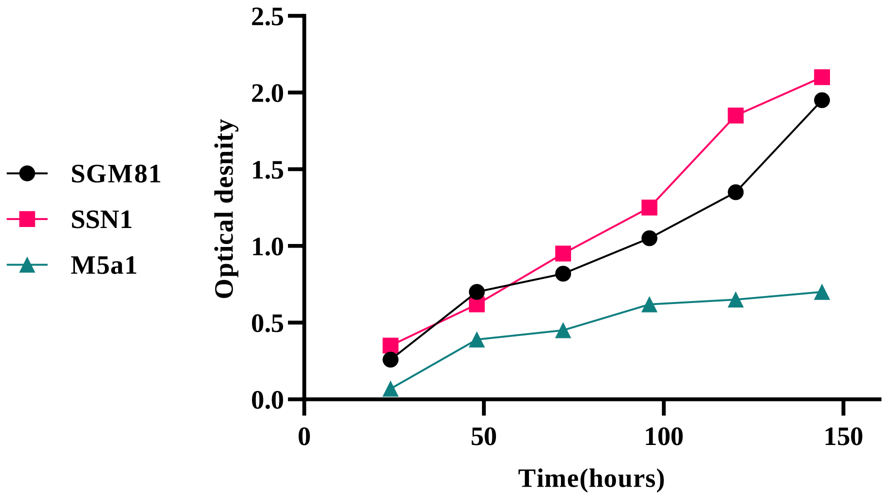

Supplementary Figure 1: Optical density of three strains, SGM81 *Klebisella quasipneumoniae*, M5a1 *Klebsiella oxytoca*, SSN1 *Klebsiella pneumonia*. The growth curve represents an increase in cell density of each isolate in response to incubation time and Jensen media.

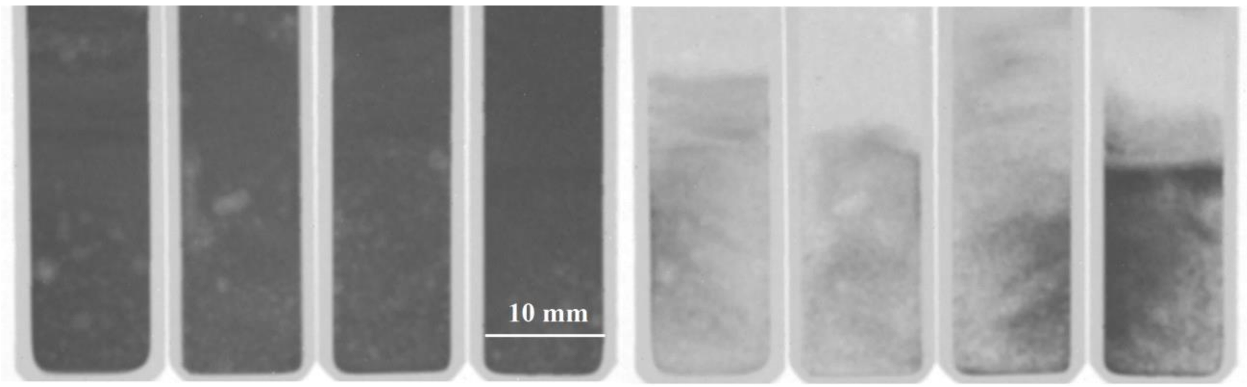

(a) (b)

Supplementary Figure 2: Neutron radiograph of soil samples at (a) t = 0 and (b) t = 24 hrs. The samples are arranged as (from left to right) control (water), M5a1, SGM81, and SSN1

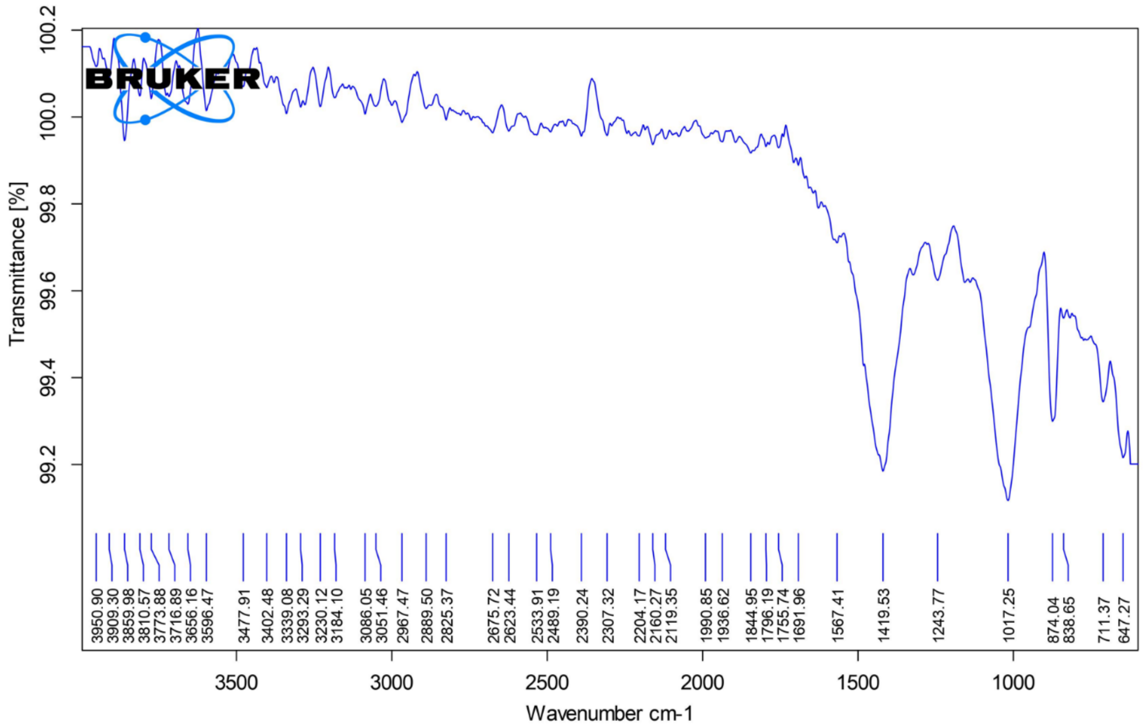

A: FTIR spectrum profile of EPS extracted in control condition

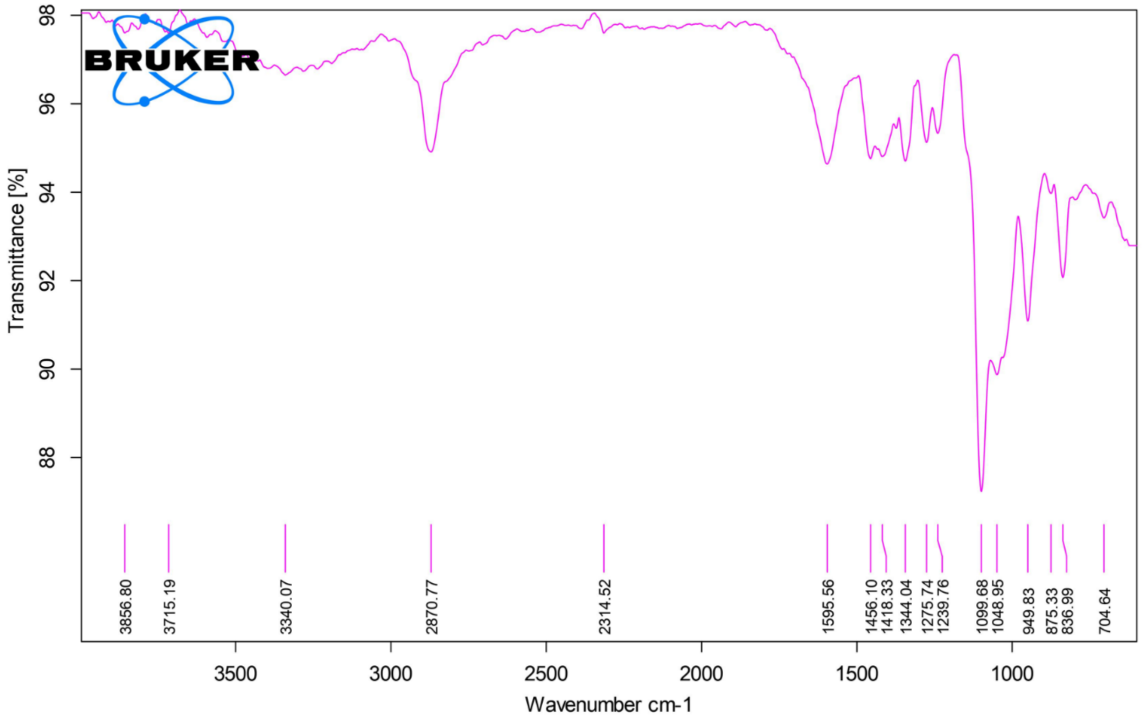

B: FTIR spectrum profile of EPS extracted in stress condition

Supplementary Figure 3:FTIR spectrum demonstrating the different wave patterns for EPS samples

Supplementary Table 1: Protein and polysaccharide concentration synthesised by SSN1 in stress and control conditions for their respective run in BBD.

Biomolecule	Run	Sucrose	% of Nitrogen	$CaCO_3$	Control %	Osmotic Stress %
Protein	3	5	0.5	0.3	0.9527 ± 0.03311	1.220 ± 0.09074
	5	1	0.1	0.1	0.7553 ± 0.06064	0.8033 ±0.07356
	6	1	0.1	0.1	0.8100 ± 0.05058	1.497 ± 0.1812
Carbohydrate	3	5	0.5	0.3	22.53 ± 0.5487	21.17 ±0.7265
	5	1	0.1	0.1	16.67 ± 0.4410	18.83 ± 0.6009
	6	1	0.1	0.1	19.50 ± 0.5774	23.67 ± 0.8413
